# Current and former smokers among adolescents aged 12–17 years in Iran: a systematic review and meta-analysis

**DOI:** 10.1186/s12889-020-8255-2

**Published:** 2020-01-31

**Authors:** Elham Ehsani-Chimeh, Haniye Sadat Sajadi, Meysam Behzadifar, Maryam Aghaei, Afsaneh Badrizadeh, Masoud Behzadifar, Nicola Luigi Bragazzi

**Affiliations:** 1 National Institute for Health Research, Tehran University of Medical Sciences, Tehran, Iran; 2 Health Management and Economics Research Center, Iran University of Medical Sciences, Tehran, Iran; 3 Department of Medicine, School of Medicine, Iran University of Medical Science, Tehran, Iran; 4 Social Determinants of Health Research Center, Lorestan University of Medical Sciences, Khorramabad, Iran; 5 Department of Health Sciences (DISSAL), Postgraduate School of Public Health, University of Genoa, Genoa, Italy; 6 Department of Mathematics and Statistics, Laboratory for Industrial and Applied Mathematics (LIAM), York University, Toronto, Canada

**Keywords:** Smoking behavior, Adolescents, Iran, Systematic review, Meta-analysis

## Abstract

**Background:**

Smoking, especially among adolescents, is considered a serious public health concern worldwide being associated with increased mortality. The present study was designed as the first systematic review and meta-analysis of the prevalence of current and former smoking behavior among adolescents in Iran.

**Methods:**

Seven international scholarly databases, namely Scopus, Embase, Pubmed/Medline, ISI/Web of Science (WOS), the Cochrane Library, Psyc Info and Cinahl, were extensively searched from January 2000 to September 18, 2019. Google Scholar was also mined. Iranian databases were searched as well (namely, MagIran, Scientific Information Database (SID), and Barakatkns). The DerSimonian-Laird’s approach, via the Freeman-Tukey double arcsine method, was used to synthesize the prevalence estimates.

**Results:**

The prevalence of current smokers among Iranian adolescents was estimated to be 9% (95% CI: 7 to 10). Stratifying based on gender, the prevalence was 12% among boys (95% CI: 10 to 14) and 6% among girls (95% CI: 5 to 8). The prevalence of former smokers among Iranian adolescents using the random-effect model was computed to be 24% (95% CI: 21 to 27).

**Conclusion:**

The findings of this study showed that the prevalence of current and former smoking behavior among Iranian adolescents is a relevant public health concern. The country’s young population should be given more attention by health policy- and decision-makers and implementation of ad hoc prevention and control policies should be on their agenda.

## Background

Smoking is considered as a serious public health concern worldwide and many healthcare planners are making serious efforts in order to reduce cigarette consumption by designing and implementing appropriate strategies in order to control one of the most important factors associated with increased mortality [1, 2]. Smoking leads, indeed, to a higher risk of cardiovascular disease, lung disorders as well as several malignancies, including lung, throat, stomach, and bladder cancers [3].

In 2010, smoking accounted for up to 6.3% of the global burden of disease [4]. In recent decades, smoking has been on the rise in developing countries, and, only after adopting appropriate policies, it has begun to slightly decline [5, 6]. The World Health Organization (WHO) has computed that approximately 7 million smokers die each year because of smoking and 1.2 million die from passive cigarette exposure [7].

Adolescents as one of the high-risk groups starting to smoke are a very important age group and special attention should be paid to their behaviors from health policy- and decision-makers [8]. Early identifying smokers in this group and the associated determinants of smoking behavior can help reduce or stop cigarette consumption [9]. Also, controlling and preventing smoking can moderate other high-risk behaviors associated with a high-risk personality, such as alcohol use, unsafe sex [10, 11] and drug use [12]. There are several factors that influence smoking in this age group. Parental smoking, economic, social and cultural status, psychological factors such as parental divorce, and gender are among the major determinants [13]. Smoking and socializing with smokers are also some of the factors that make adolescents more likely to continue smoking [14].

Iran is one of the countries characterized by a young population. It is important for health planners to monitor their behaviors and try to keep cigarette consumption low among this age group, implementing ad hoc prevention and control programs if needed. Short- and long-term tobacco control plans can have many potential benefits in terms of health.

In recent years, several studies have been conducted on the prevalence of current and former smoking behavior among Iranian adolescents. Updated information can be used as valuable evidence for tobacco-related policies as well as for future chronic disease burden calculation. Health managers and decision-makers can rely on comprehensive reviews to design policies to prevent and control smoking among adolescents. To the best of our knowledge, there exists no a comprehensive synthesis of cigarette consumption rate in Iran. Therefore, the present study was designed as the first systematic review and meta-analysis of the prevalence of current and former smokers in this age group in Iran, in order to fill this gap in knowledge.

## Methods

### Literature search and review

Seven international scholarly databases, namely Scopus, Embase, Pubmed/Medline, ISI/Web of Science (WOS), the Cochrane Library, PsycInfo and Cinahl, were extensively searched from January 2000 to September 18, 2019. Google Scholar was also mined to increase the chance of finding potentially relevant studies related to the topic under scrutiny.

Iranian databases were searched as well (namely, MagIran, Scientific Information Database (SID), and Barakatkns). To systematically retrieve articles on cigarette consumption among Iranian adolescents, a string of keywords was used in the English or Farsi languages: Boolean operators (OR, AND, NOT) were used to properly connect the various terms. In particular, the search strategy was the following: Iran AND (tobacco OR cigarette OR cigarettes OR smoke OR “smoking behavior” OR “smoking”) AND (youth OR adolescent OR adolescents OR student OR students) AND (epidemiology OR frequency OR rate OR prevalence OR use). The reference list of potentially relevant studies was also scanned for cross-referencing purpose.

Two authors independently conducted the literature search and review. The disagreement between them was resolved involving a third author, if necessary, or through discussion until agreement was achieved.

### Primary outcomes

The primary outcomes of the present systematic review and meta-analysis were: i) the prevalence of current smokers, ii) the prevalence of former smokers, and, iii) the determinants of smoking behavior. The following definitions were adopted: a smoker was considered a current or former smoker if is smoking daily or if has quit smoking in the past 30 days.

### Inclusion and exclusion criteria

Studies with the following characteristics were selected if: i) published in the English or Farsi languages, ii) conducted in the period 2000 to September 2019, iii) published in peer-reviewed journals, iv) providing sufficient data to calculate the prevalence of current and former smokers among Iranian adolescents aged 12–17 years, v) designed as cross-sectional investigations, and vi) questionnaire-based.

The following studies were excluded if: i) conducted before 2000, ii) not available as full-text, iii) not been peer-reviewed and published as abstract/conference proceedings, iv) lacking quantitative details, v) utilizing small sample sizes (less than 50 participants), vi) exploring the use of electronic cigarettes, vii) designed as case-reports, case-series, case-control or randomized clinical trials (RCTs), and viii) not being conducted in Iran.

Two authors independently selected studies based on the above-mentioned inclusion and exclusion criteria.

### Data extraction

The relevant data extracted included: surname of the first author, year of publication, country area/province of the study, sample size, mean age of participants, tool used to investigate current and former cigarette consumption, prevalence reported in the study, gender, study design, and determinants of smoking behavior. Two authors independently extracted the data utilizing an ad hoc designed, structured Excel spreadsheet. The disagreement between the two authors regarding the data extraction process was resolved through discussion.

### Methodological quality assessment

The Joanna Briggs Institute (JBI) checklist for analytical cross-sectional studies was used to critically evaluate the quality of the methodology of the selected studies [15]. This checklist contains 8 questions with four possible replies (“yes”, “no”, “unclear”, “not applicable”), exploring if: i) inclusion criteria are well defined, ii) study subjects and the setting are clearly described in detail, iii) exposure is measured in a valid, standardized, reliable way, iv) the condition is measured in an objective, standardized fashion, v) confounding factors are clearly identified and vi) properly corrected/adjusted for, with strategies clearly stated, vii) outcomes are properly measured, and viii) statistical analyses used are appropriate.

The Cohen’s Kappa coefficient was used for measuring the degree of disagreement between the two independent authors during all the steps of the systematic review (search literature, studies selection, data extraction and methodological quality assessment of the studies) [16].

### Statistical analysis

The DerSimonian-Laird approach, via the Freeman-Tukey double arcsine method, was used in order to estimate the pooled prevalence of current and former smokers among Iranian adolescents [17]. All synthesized estimates were reported with their computed 95% confidence interval (CI). *p*-value < 0.05 was considered significant. Initially, a preliminary analysis was performed and if heterogeneity among studies was over 50% a random-effect model was preferred to a fixed-effect model to analyze all the data. In more detail, the I^2^ statistic was used to evaluate the amount of heterogeneity [18], which, depending on the I^2^ value, was stratified into low (25%), moderate (50%) and high (75%). Publication bias was assessed inspecting the funnel plot in terms of asymmetry and using the Egger’s linear regression test [19]. Sensitivity analysis was conducted to evaluate the data stability. The odds ratio (OR) was computed to compare the risk of smoking behavior based on the gender. Subgroup-analysis was performed to investigate possible sources of heterogeneity. For each moderator variable, a meta-regression analysis was also carried out according to the year of data collection and sample size to estimate their impact of the prevalence of current and former smokers. Data were analyzed using the STATA Ver.12 (Stata Corp, College Station, TX, USA) software.

## Results

This study adhered to the “Preferred Reporting Items for Systematic Reviews and Meta-Analyses” (PRISMA) guidelines [20].

**Fig. 1:**
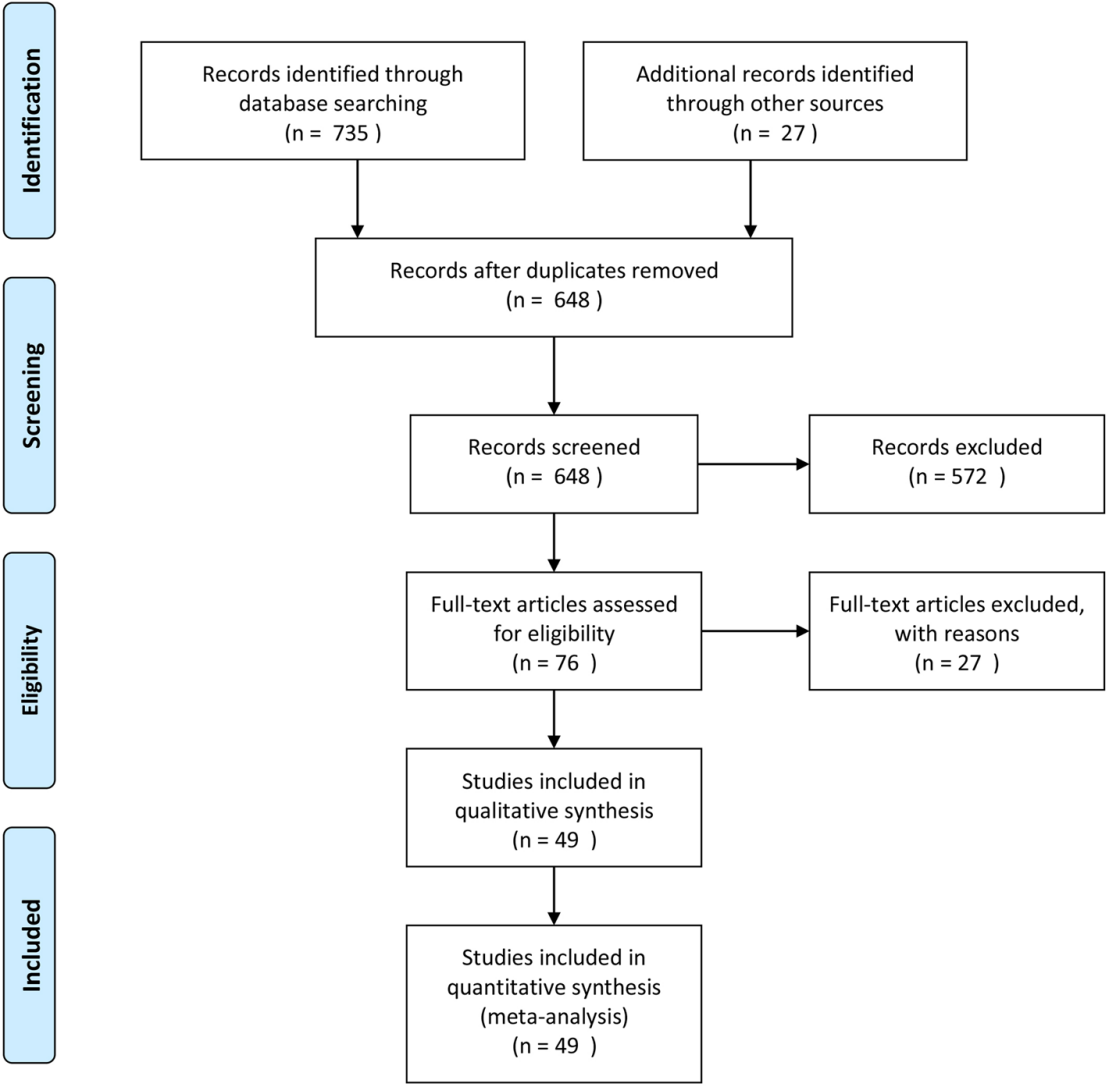
Flow-chart showing the process of study retrieval, selection and inclusion adopted in the present systematic review and meta-analysis

An initial literature search according to our search strategy resulted in a pool of 762 articles. We excluded 114 articles, because they were duplicate items. After scrutinizing the titles and/or abstracts, 572 articles were excluded. The full text of 76 articles was screened in-depth and, finally, according to the previously mentioned inclusion and exclusion criteria, 49 articles were retained in the present systematic review and meta-analysis [21–69]. The entire process of study retrieval and selection is pictorially shown in Fig. 1**.**

The included studies were conducted between 2004 and 2019, investigating a total sample of 71,859 Iranian adolescents. All the selected studies were cross-sectional investigations. The main characteristics of the selected studies are shown in Table 1, whereas the results of their methodological quality assessment are reported in Table 2.

**Table 1:** Characteristics of included studies

First author	Year	City	Mean age ± SD	Sample size	No.boy	No. girl	Tools	Geographical area	Where adolescents live (urban, rural)
Ayatollahi	2004	Shiraz	16 ± 0.77	1132	1132	0	SRQ	East	Urban
Kelishadi	2004	Isfahan	NA	1950	946	1004	SRQ	Central	Both
Mojahed	2004	Zahedan	15.9 ± 1	475	216	259	SRQ	Central	Urban
Najafi	2005	Rasht	NA	1474	751	723	SRQ	Central	Urban
Vafaei	2005	Tabriz	NA	1000	NA	NA	SRQ	West	Urban
Heydari	2007	Tehran	16.7	1093	712	381	GYTS	West	Urban
Najafi	2007	Guilan	NA	1927	1041	886	SRQ	North	Urban
Barikani	2008	Tehran	14.8 ± 1.4	700	374	326	SRQ	Central	Urban
Namakin	2008	Birjand	16.3 ± 1.3	1233	1233	0	SRQ	Central	Urban
Ziaaddini	2008	Kerman	NA	860	346	514	SRQ	East	Urban
Mohtasham Amiri	2009	Rasht	16.2 ± 0.9	1400	1400	0	SRQ	North	Urban
Charkazi	2010	Zahedan	15.9 ± 7	380	NA	NA	SRQ	Central	Urban
Emami	2010	Tehran	17.53 ± 0.59	4566	2069	2497	SRQ	North	Urban
Gharlipour Gharghani	2010	Shiraz	NA	244	244	0	SRQ	Central	Urban
Grarmaroudi	2010	Tehran	16	2400	1200	1200	SRQ	North	Urban
Moeini	2010	Malayer	NA	900	900	0	SRQ	West	Urban
Mohammadpoorasl	2010	Tabriz	16.28 ± 0.87	1785	NA	NA	SRQ	West	Urban
Pasharavesh	2010	Kermanshah	16.36 ± 1.17	3150	0	3150	GYTS	West	Urban
Rahmanian	2010	Jahrom	NA	1145	697	448	SRQ	South	Both
Ramezankhani	2010	Tehran	14.69 ± 2.09	4523	2272	2251	SRQ	Central	Urban
Alaee	2011	Karaj	16.5 ± 1.299	447	208	239	GYTS	Central	Urban
Hamidzade Arbaby	2011	Khalkhal	NA	260	260	0	SRQ	West	Urban
Ghavidel	2012	Nazarabad	17	400	204	196	SRQ	North	Urban
Mohammadkhani	2012	10 provinces	NA	2538	1283	1255	SRQ	NA	Urban
Heydari	2013	Tehran	NA	1271	1271	0	SRQ	Central	Urban
Javadzade	2013	Isfahan	17.72 ± 0.62	382	382	0	SRQ	Central	Urban
Karimi	2013	Zarandieh	16.21 ± 1.45	250	250	0	SRQ	East	Urban
Nazarzadeh	2013	Zanjan	17.2 ± 1.3	352	352	0	SRQ	East	Urban
Barati	2014	Hamadan	16.42 ± 0.89	810	810	0	SRQ	West	Urban
Bidel	2014	Ilam	16.2 ± 0.5	1000	1000	0	SRQ	West	Urban
Esmaielzadeh	2014	Qazvin	NA	510	271	239	SRQ	West	Urban
Khajehdaluee	2014	Sarakhs	NA	943	507	436	SRQ	West	Both
Miri	2014	Birjand	17.024 ± 0.89	2371	2371	0	SRQ	East	Urban
Mohammadi	2014	Babolsar	15.3 ± 0.5	450	450	0	SRQ	North	Both
Pirdehghan	2014	Yazd	16.02 ± 0.9	460	273	187	GYTS	East	Urban
Chaman	2015	Shahroud	16.5 ± 1.1	450	450	0	SRQ	East	Urban
Madani	2015	Bandar Abbas	16 ± 1.34	2029	1009	1020	SRQ	South	Urban
Meysamie	2015	Tehran	16.21	2877	1557	1320	SRQ	Central	Urban
Rashid	2015	Tehran	16.5	1022	511	511	SRQ	Central	Urban
Kelishadi	2016	30 provinces	12.47 ± 3.36	13,486	6846	6640	GSHS	NA	Both
Khoramdad	2016	Shiraz	17	750	569	181	SQR	Central	Urban
Fakharri	2017	Tabriz	NA	1000	460	540	MCMI-III	West	Urban
Karimi	2017	Shiraz	16.11 ± 1.16	842	842	0	SRQ	Central	Urban
Mohamadzadeh	2017	Ilam	NA	372	199	173	SRQ	West	Urban
Mohammadi	2017	Marivan	16.2 ± 0.25	470	470	0	SRQ	West	Urban
Ataeiasl	2018	Tabriz	15.48 ± 0.50	1133	567	566	SRQ	West	Urban
Farshidi	2018	Bandar Abbas	16.4 ± 1.1	422	422	0	SRQ	South	Urban
Jamshidi	2018	Ahvaz	16.56 ± 15.02	899	450	449	SRQ	South	Urban
Ansari	2019	Zahedan	NA	1094	613	481	SRQ	East	Urban

Global youth tobacco *survey* (*GYTS*), Self-report questionnaire (*SRQ*), Global School-based Student Health Survey (*GSHS*), Millon Clinical Multiaxial Inventory (*MCMI-III*), Not available (*NA*)

**Table 2:** Results of quality assessment

First author	Year	Q1	Q2	Q3	Q4	Q5	Q6	Q7	Q8
Ayatollahi	2004	Yes	No	Yes	No	Yes	Yes	Yes	Yes
Kelishadi	2004	Yes	Yes	Yes	Yes	Yes	Yes	No	Yes
Mojahed	2004	Yes	Yes	Yes	Yes	Yes	Yes	Yes	Yes
Najafi	2005	Yes	Yes	Yes	Yes	No	Yes	No	Yes
Vafaei	2005	Yes	No	Yes	Yes	Yes	Yes	Yes	Yes
Heydari	2007	Yes	Yes	No	Yes	Yes	Yes	Yes	Yes
Najafi	2007	Yes	No	Yes	Yes	No	Yes	Yes	Yes
Barikani	2008	Yes	Yes	Yes	Yes	Yes	No	Yes	Yes
Namakin	2008	Yes	Yes	Yes	Yes	Yes	Yes	Yes	Yes
Ziaaddini	2008	Yes	No	Yes	No	Yes	Yes	Yes	Yes
Mohtasham Amiri	2009	Yes	Yes	Yes	Yes	Yes	No	Yes	Yes
Charkazi	2010	Yes	No	Yes	Yes	Yes	Yes	Yes	Yes
Emami	2010	Yes	Yes	No	Yes	No	Yes	Yes	Yes
Gharlipour Gharghani	2010	Yes	No	Yes	Yes	Yes	Yes	No	Yes
Grarmaroudi	2010	Yes	Yes	Yes	Yes	Yes	No	Yes	Yes
Moeini	2010	Yes	No	Yes	Yes	No	Yes	Yes	Yes
Mohammadpoorasl	2010	Yes	Yes	Yes	Yes	Yes	Yes	No	Yes
Pasharavesh	2010	Yes	Yes	Yes	No	Yes	Yes	Yes	Yes
Rahmanian	2010	Yes	No	Yes	Yes	Yes	No	Yes	Yes
Ramezankhani	2010	Yes	No	No	Yes	Yes	Yes	Yes	Yes
Alaee	2011	Yes	Yes	Yes	Yes	Yes	Yes	Yes	Yes
Hamidzade Arbaby	2011	Yes	Yes	Yes	Yes	Yes	No	Yes	Yes
Ghavidel	2012	Yes	Yes	Yes	Yes	Yes	Yes	Yes	Yes
Mohammadkhani	2012	Yes	No	Yes	No	Yes	Yes	Yes	Yes
Heydari	2013	Yes	No	Yes	Yes	Yes	No	Yes	Yes
Javadzade	2013	Yes	Yes	Yes	Yes	Yes	Yes	No	Yes
Karimi	2013	Yes	Yes	Yes	Yes	Yes	Yes	Yes	Yes
Nazarzadeh	2013	Yes	Yes	Yes	No	Yes	Yes	Yes	Yes
Barati	2014	Yes	Yes	Yes	Yes	Yes	No	Yes	Yes
Bidel	2014	Yes	Yes	Yes	Yes	Yes	No	Yes	Yes
Esmaielzadeh	2014	Yes	No	Yes	Yes	No	Yes	Yes	Yes
Khajehdaluee	2014	Yes	No	Yes	Yes	Yes	Yes	Yes	Yes
Miri	2014	Yes	Yes	Yes	Yes	Yes	Yes	No	Yes
Mohammadi	2014	Yes	Yes	No	Yes	Yes	No	Yes	Yes
Pirdehghan	2014	Yes	Yes	Yes	Yes	Yes	Yes	Yes	Yes
Chaman	2015	Yes	Yes	Yes	No	Yes	Yes	Yes	Yes
Madani	2015	Yes	Yes	No	Yes	Yes	No	Yes	Yes
Meysamie	2015	Yes	Yes	Yes	Yes	Yes	Yes	No	Yes
Rashid	2015	Yes	Yes	Yes	Yes	No	Yes	Yes	Yes
Kelishadi	2016	Yes	Yes	Yes	No	Yes	Yes	No	Yes
Khoramdad	2016	Yes	Yes	Yes	Yes	Yes	No	Yes	Yes
Fakharri	2017	Yes	No	Yes	Yes	Yes	Yes	No	Yes
Karimi	2017	Yes	Yes	Yes	Yes	Yes	Yes	Yes	Yes
Mohamadzadeh	2017	Yes	Yes	Yes	Yes	Yes	Yes	Yes	Yes
Mohammadi	2017	Yes	Yes	No	Yes	Yes	Yes	No	Yes
Ataeiasl	2018	Yes	Yes	Yes	Yes	Yes	Yes	Yes	Yes
Farshidi	2018	Yes	Yes	Yes	Yes	Yes	Yes	No	Yes
Jamshidi	2018	Yes	Yes	Yes	Yes	No	Yes	Yes	Yes
Ansari	2019	Yes	No	Yes	Yes	Yes	Yes	No	Yes

(Q1: Were the criteria for inclusion in the sample clearly defined?, Q2: Were the study subjects and the setting described in detail?, Q3: Was the exposure measured in a valid and reliable way?, Q4: Were objective, standard criteria used for measurement of the condition?, Q5:Were confounding factors identified?, Q6: Were strategies to deal with confounding factors stated?, Q7: Were the outcomes measured in a valid and reliable way?, Q8: Was appropriate statistical analysis used?)

### Current cigarette smokers

#### The prevalence of current smokers

**Fig. 2:**
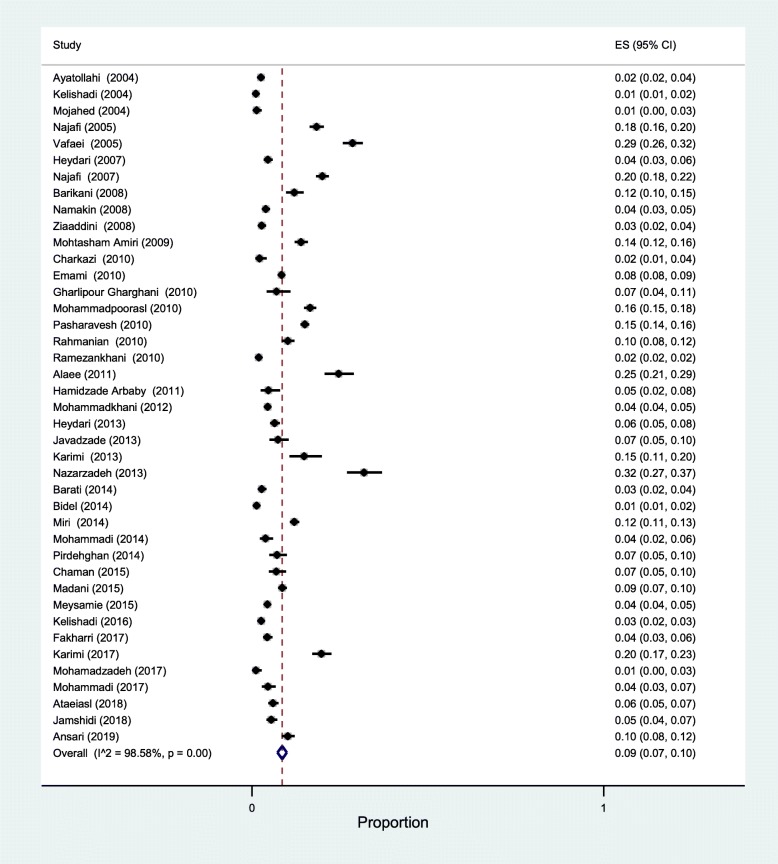
The forest plot of the studies included in the present systematic review and meta-analysis investigating the prevalence of current smoking behavior among Iranian adolescents using the random-effect model

Forty-one studies reported participants’ current smoking status. The overall prevalence of current smokers among Iranian adolescents was estimated to be 9% (95% CI: 7 to 10) with a highly, statistically significant heterogeneity (I^2^ = 98.58%). (Fig. 2). Stratifying based on gender, the prevalence was 12% among boys (95% CI: 10 to 14) and 6% among girls (95% CI: 5 to 8) (Appendix 1).

Sensitivity analysis was performed, and the results did not change before and after the analysis, indicating consistent results. There was no evidence of publication bias according to the visual inspection of the funnel plot and the Egger’s linear regression test (*p* = 0.643).

**Fig. 3:**
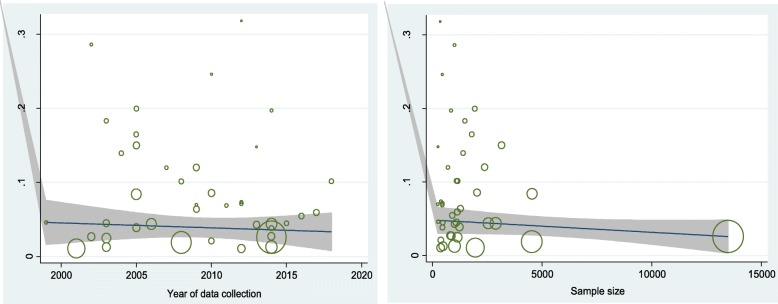
Results of meta-regression analysis based on the year of data collection and sample size of the included studies of current smoking behavior among Iranian adolescents

Meta-regression was performed based on the year of data collection and sample size. The results showed that both these variables had no impact on the prevalence of current smokers among adolescents (*p* = 0.554 and *p* = 0.397, respectively) (Fig. 3).

The findings of the sub-group analyses based on gender and year of data collection are shown in Table 3. The prevalence among boys displayed a slightly increase throughout the time, whereas a slightly decreasing trend was found among girls. The prevalence of current smokers was higher in boys than girls. However, these findings should be interpreted with caution and only as suggestive of trends, in that: i) the sample sizes are relatively small, and ii) the 95%CIs are quite wide and partially overlapping.

**Table 3:** Sub-group analyses based on gender and publication year for current smokers among Iranian adolescents

Gender	Time Period (year)	Prevalence (95% CI)	I^2^	*P*-value
Male	2000–2005	10% (2 to 19)	98.74%	0.38
	2006–2010	10% (6 to 15)	98.73%	0.12
	2011–2015	11% (8 to 15)	97.82%	0.72
	2016–2019	19% (8 to 31)	99.53%	0.46
Female	2000–2005	5% (4 to 13)	98.6%	0.19
	2006–2010	7% (4 to 11)	99.13%	0.52
	2011–2015	5% (2 to 7)	94.73%	0.82
	2016–2019	6% (3 to 8)	98.63%	0.28

**Fig. 4:**
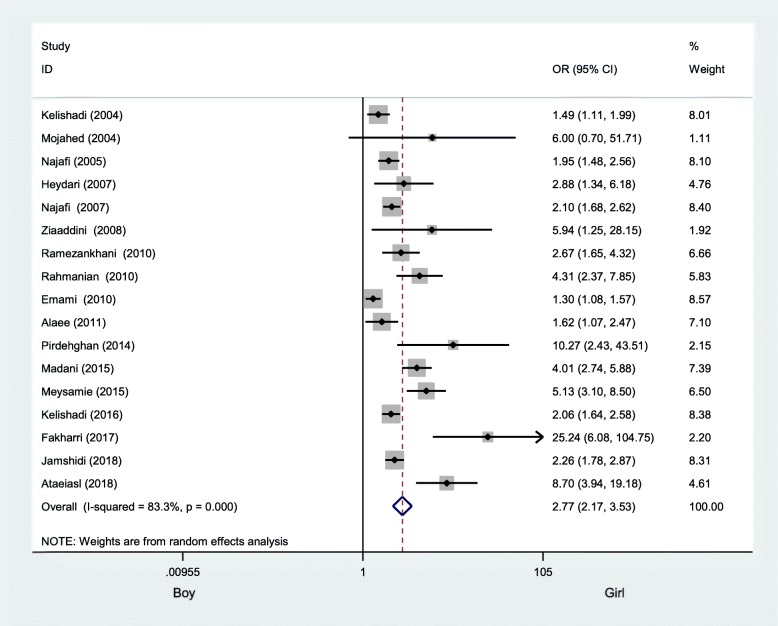
Odds ratio of current smoking behavior among Iranian boys compared to girls using the random-effect model

Boys had a higher risk of being current smokers with respect to girls, with an OR of 2.77 (95% CI: 2.17 to 3.53) (Fig. 4).

The prevalence of current smoking behavior stratified by geographical area and tool used is presented in Table 4, showing no effect of both variables.

**Table 4:** Prevalence rate of current smoking behavior stratified according to geographical area and tool used

Variable	Prevalence 95% CI	I^2^
Tools		
GYTP	7% (1 to 13)	99.25%
MCMI-III	4% (3 to 6(	–
GSHS	3% (2 to 3)	–
SRG	9% (7 to 11)	98.48%
Geographical area		
Central	7% (5 to 9)	97.47%
East	10% (6 to 13)	97.73%
North	13% (7 to 18)	98.48%
South	8% (5 to 11)	88.82%
West	8% (4 to 12)	98.97%

Among the included studies, 16 studies recruited only boys and the prevalence of current smokers was estimated to be 9% (95% CI: 6 to 11) with I^2^ = 97.6%. Only the study by Pasharavesh et al. utilized a sample of girls [38]. A further 24 studies reported current smoking status in both genders and the prevalence of current smokers was estimated to be 8% (95% CI: 7 to 10) with I^2^ = 98.8%.

### Former cigarette smokers

#### The prevalence of former smokers

**Fig. 5:**
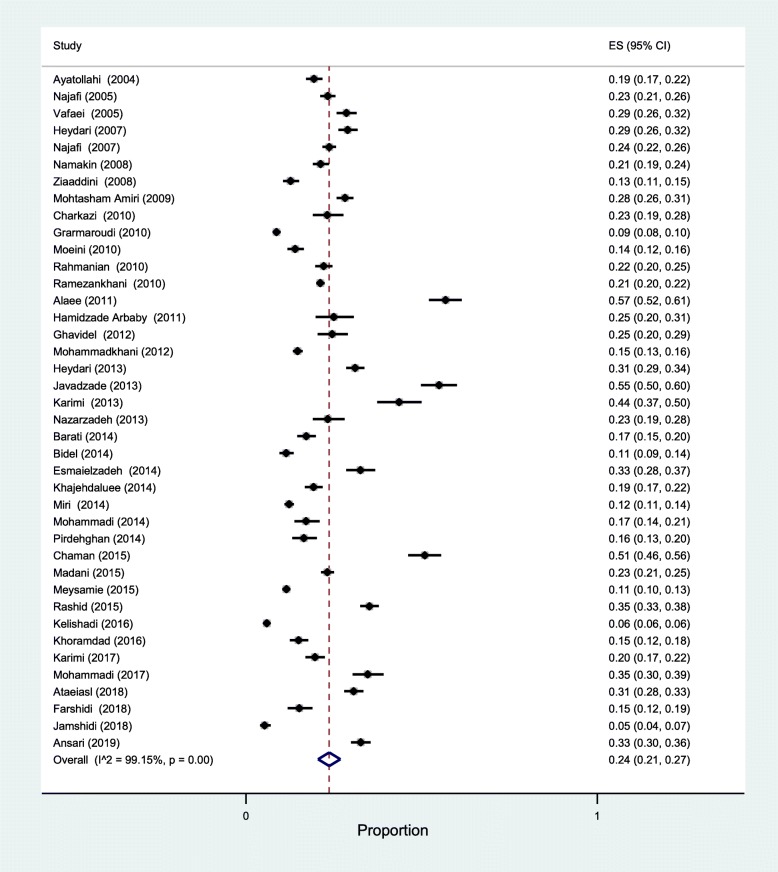
The forest plot of the studies included in the present systematic review and meta-analysis investigating the prevalence of former smoking behavior among Iranian adolescents using the random-effect model

40 studies reported participants’ former smoking status. The prevalence of former smokers among Iranian adolescents using the random-effect model was computed to be 24% (95% CI: 21 to 27) with a high, statistically significant heterogeneity (I^2^ = 99.15) (Fig. 5).

Publication bias visually inspecting the funnel plot and using the Egger’s linear regression test was not detected (*p* = 0.12). Also, sensitivity analysis showed no changes before and after analysis and confirmed the consistency of results.

**Fig. 6:**
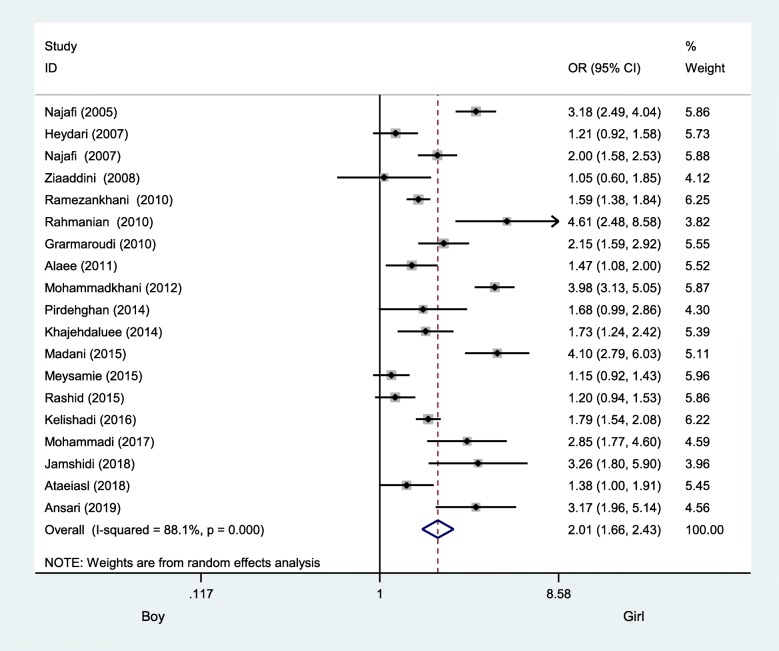
Odds ratio of former smoking behavior among Iranian boys compared to girls using the random-effect model

The prevalence of former smoking behavior among boys was 25% (95% CI: 21 to 28), with an amount of heterogeneity of I^2^ = 98.81%, and among girls was 12% (95% CI: 10 to 15), with an amount of heterogeneity of I^2^ = 97.89% (Appendix 2). Boys had a higher former smoking behavior risk with respect to boys, with an OR of 2.01 (95% CI: 1.66 to 2.43) (Fig. 6).

Sub-analyses of prevalence of former smoking behavior stratified by gender and year of data collection are presented in Table 5.

**Table 5:** Sub-group analyses based on gender and publication year for former smokers among Iranian adolescents

Gender	Time Period (year)	Prevalence Rate (95% CI)	I^2^	P-value
Male	2000–2005	32% (7 to 68)	99.32%	0.61
	2006–2010	21% (15 to 26)	98.01%	0.35
	2011–2015	29% (23 to 36)	98.62%	0.24
	2016–2019	17% (11 to 23)	98.87%	0.18
Female	2000–2005	14% (12 to 17)	–	0.16
	2006–2010	11% (6 to 17)	97.90%	0.57
	2011–2015	18% (11 to 24)	98.43%	0.48
	2016–2019	7% (4 to 10)	91.82%	0.71

**Fig. 7:**
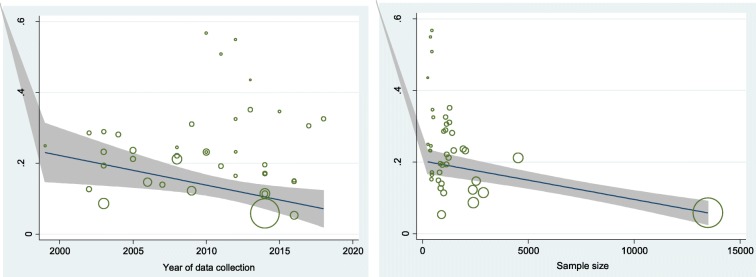
Results of meta-regression analysis based on the year of data collection and sample size of the included studies of former smoking behavior among Iranian adolescents

Meta-regression was performed based on the year of data collection and sample size, showing no impact (*p* = 0.798 and *p* = 0.281, respectively) (Fig. 7).

Among the included studies, 20 studies recruited only boys and the prevalence of former smokers was estimated to be 25% (95% CI: 20 to 29) with I^2^ = 99.3%. Only the study of Pasharavesh et al. was conducted utilizing a sample of girls [38]. A further 19 studies reported former smoking status in both genders and the prevalence of former smokers was estimated to be 23% (95% CI: 19 to 27) with I^2^ = 98.3%.

## Discussion

The findings of the present study showed that the prevalence of current smoking behavior among Iranian adolescents was 9%. This was lower than the prevalence of cigarette consumption among Iranian adults (14.38%) [70], and among college students (23.8%) [71], as reported in the existing scholarly literature. Compared to international studies, our results are lower than those found in studies conducted in Saudi Arabia (19.5%) [72], India (11.8%) [73], Sudan (13.6%) [74] and Italy (35.6%) [75] and higher than the prevalence computed in studies carried out in Ethiopia (3%) [76] and China (7.93%) [77]. Our estimates were similar to the results of a meta-analysis conducted in East Africa (9.02%) [78]. The prevalence of former smoking behavior among Iranian adolescents was estimated to be 24%. This rate was lower than the findings of studies carried out in Mexico (29.6%) [79] and Nigeria (32%) [80] but higher than the findings of a study performed in Turkey (12%) [81].

This difference in prevalence across studies can be due to social, cultural, health conditions, and legal factors [72, 74, 75, 78]. Different study settings could explain the variability of the findings: some studies were done in schools and teachers were present when the data were collected. This could have affected the reliability of the replies [75].

Both current and former smoking behavior was higher in boys than in girls [2, 5, 8, 9, 13]. Men are more at risk of exposure to cigarettes and other health problems due to social conditions, curiosity, propensity to experiencing high-risk situations, and the pressures they face [72]. In boys, the risk of smoking increases with parents (especially fathers) smoking. Parental education is, as such, very important. Women tend to smoke less because of personality traits [82] as well as, especially in countries like Iran, for cultural reasons and stigma. Unfortunately, adolescent boys, due to the high influence of their friends, see smoking as a sign of adulthood, and their lack of awareness and education has made them more likely to smoke than adolescent girls [74, 80, 81].

The results of the analysis based on geographical areas showed that the highest prevalence was in the north (13%) and east (10%), even though this should be interpreted only as a trend, given the absence of statistical significance. In the northern parts of Iran, drug use is, indeed, of great concern, and the high prevalence of drug use among parents and adolescents has made them more likely to smoke [83]. The eastern part of Iran is bordered by Afghanistan and Pakistan and, therefore, the high prevalence of narcotics in these two countries has led to a higher volume of cigarette consumption and drug use in these provinces. According to adolescents, these areas are among those with the highest prevalence of cigarette consumption in Iran [84].

Result of meta-regression according to the year of data collection in current adolescent smokers show a marked increase, although not statistically significant, but this increase can be considered as an important signal for health policy- and decision-makers. On the other hand, in recent decades, one of the factors affecting the behavior of adolescents in Iran and elsewhere in the world is the increasing access to virtual and internet networks. By creating new patterns of behaviors, these networks have induced people to adopt new lifestyles, which also include appropriate programs to deal with and prevent risk factors [85].

### Factors affecting smoking behavior in adolescents in Iran

In the studies selected, various factors were found to be associated with smoking behavior, such as the presence of a smoker in the family, a lack of awareness of the consequences of smoking, easy access to cigarettes, and the lack of governmental laws to sell them to adolescents. Deaths of family members, parents, gender, smoking friends, dissatisfaction with family, highly emotional environment, divorce, family disputes, history of school and family escape, curiosity about smoking experience, social problems, low parental education level and low family economic level were other significant determinants of smoking behavior (Appendix 3).

According to most studies, smoking was mentioned as a common habit because of its cheapness, convenience and affordability. In Iran, there are no smoking rules for people under the age of 18, in terms of rules prohibiting shops to sell them to this age group. The problem with this is the lack of control over these stores, and teens can easily buy and consume cigarettes. The low price of cigarettes in Iran has made adolescents not having a particular problem in buying cigarettes. Restrictions on access, price hikes, and stricter laws are effective in preventing and controlling smoking behavior among adolescents and should be addressed by health policy- and decision-makers. There was a significant relationship between the decrease in smoking and the increase in its price, with some adolescents being unable to afford the costs of purchasing cigarettes. Adopting rules to limit smoking in teens is expected to slow down the process of smoking [86].

Basically, smoking in Iranian society is not well-perceived because of religious and cultural issues, and there is a negative attitude towards smokers (especially women). People cannot smoke in public environments such as schools and universities. Religious issues can be widely used for prevention and control tobacco in Iran. In some selected studies, adolescents with stronger religious beliefs were less likely to smoke. Parental education is another valid strategy in that many families have been able to prevent smoking behavior among their children by raising their awareness of the consequences of being exposed to cigarette smoke [87]. Therefore, the role of religion as a deterrent to high-risk behaviors in adolescents should be considered [88] as well as the role of parental education.

Another factor contributing to the increase in cigarette consumption is their social attractiveness and availability in the market (such as, various forms of cigarettes with different shapes and colors, attractive flavors and envelopes, showing attractive scenes of popular young actors and actresses consuming them). According to some studies, the attractiveness of smoking and its widespread advertising in the stores have led teens to try to smoke as a new experience. In recent years, Iranian laws have banned smoking scenes on television, and this tobacco control policy has been very useful for prevention, even if partially.

Family disputes, divorce, and parental neglect on behaviors that put adolescents at risk have been mentioned as smoking determinants in many studies. Personality traits are also another crucial factor of smoking: anxiety, willingness to take risky behaviors [89] as well as curiosity and the tendency for new emotions and experiences. Many people smoke cigarettes because of their inability to manage their negative emotions and to avoid problems and stressors. The instant relaxation of a cigarette encourages cigarette consumption over time. In these people knowledge of emotion control skills and stress reduction strategies are very useful in preventing or, at least, reducing smoking behavior [78].

Moreover, family plays an important role in shaping adolescents’ personality, building confidence, feeling secure, and behaving in a more proactive manner. Sufficient support, attention, and sensitivity of parents can help a person adapt better to the environment, coping with problems, increasing social skills and reducing the risk of unhealthy behaviors. On the other hand, family can make the adolescent feel safe or vice versa, cause him or her to develop psychological issues [88, 90]. The more parents ignore the youth, the more vulnerable they are to the environment and society. Young people in poorly supportive families are more likely to experience anxiety, depression, low self-esteem and self-efficacy, and problems in relationships, as well as various educational and social failures. In these situations, people are more likely to resort to smoking, alcohol, or drugs to get rid of their problems. In addition, parents are the most effective example in terms of behavioral patterns for their children [73].

The impact of friends and peers in terms of adoption of particular attitudes and behaviors such as the tendency to smoke was also highly emphasized in many studies. Many people start smoking imitating their peers. Some adolescents can give up on their requests and wishes, because they want to be in touch with their friends and peers, since it is so important to be accepted by friends during the periods of adolescence and young adulthood [72]. These behaviors can be exacerbated when one wants to define their identity by communicating with their favorite friendship group. Under these circumstances, peers are more likely to be accepted if smoking [91].

Furthermore, in most studies included in the present systematic review and meta-analysis, many adolescents did not know much about the effects of smoking. They were not educated by their families and teachers about the consequences of being exposed to cigarette smoke. Although many health professionals regularly report on the harm of smoking, many people are unaware of the exact side effects of smoking [92]. Having information about the annual deaths from smoking and the number of people with lung, breast, skin, bladder cancers or other tumors caused by smoking can be more effective in preventing cigarette consumption. The lack of this information leads to the formation of false beliefs about smoking and to the fact that people do not take the effects of smoking seriously and think that severe illnesses such as cancers are rare [93].

Moreover, other factors influencing smoking behavior among adolescents in many of the studies selected were economic conditions and parental literacy. Families whose parents had a low economic level were more likely to smoke and this effect on adolescents’ behaviors was particularly evident in some selected studies. In addition, adolescents whose parents had low literacy levels were more likely to smoke, which was consistent with findings from other studies carried out in various countries [74, 82, 92].

### Limitations

This study has some limitations that may be mentioned. The high, statistically significant heterogeneity found is the major shortcoming. Further, countrywide studies conducted using standardized, validated questionnaires with a large sample size could not be found in the present systematic review and meta-analysis. This should be on the agenda of healthcare decision-, policy-makers and researchers for the future. Moreover, the study data collected were insufficient to investigate the relationship between smoking determinants and the prevalence of current/former smoking behavior from a quantitative standpoint. As such, we could only perform a qualitative synthesis of tobacco-related predictors. Finally, in some provinces and regions of Iran, no studies have been conducted so far on the topic object of the present investigation.

## Conclusion

The findings of this study showed that the prevalence of current smoking behavior among Iranian adolescent boys is rather high and has been increasing throughout the time, even though not in a statistically significant way. The country’s young population should be given more attention by health policy- and decision-makers. Implementation of ad hoc prevention and control policies should be on their agenda. The cooperation of families and teachers plays a very important role with this regard. If these policies are not implemented, the Iranian health sector will face serious problems with the consequences of smoking. Raising awareness and providing appropriate conditions to reduce risky behaviors among adolescents can greatly prevent their propensity to smoke.

## Data Availability

Not applicable.

## Appendix 1

Figure 8 The prevalence of current smokers according to gender among adolescents aged 12 – 17 years in Iran (boys, 1A, and girls, 1B). a The prevalence of current smokers among Iranian boys aged 12 – 17 years. b The prevalence of current smokers among Iranian girls aged 12 – 17 years

## Appendix 2

Figure 9 The prevalence of former smoking behavior according to gender among adolescents aged 12 – 17 years in Iran (2A, boys, and girls, 2B). a The prevalence of former smoking behavior among Iranian boys aged 12 – 17 years. b The prevalence of former smoking behavior among Iranian girls aged 12 – 17 years

## Appendix 3

Table 6 Factors affecting smoking behavior according to the studies included in the present systematic review and meta-analysis

**Table:**

Factors affecting smoking behavior in included studies	Number of studies per factor	Number of studies reporting a positive strength of the factor significantly affecting smoking behavior
Smoker in the family	27 (55.1%)	8 (29.6%)
Lack of awareness of the consequences of smoking	16 (32.7%)	5 (31.3%)
Easy access to cigarettes	14 (28.6%)	4 (28.6%)
The lack of governmental laws to sell cigarettes to adolescents	3 (6.1%)	0 (0.0%)
Deaths of family members	9 (18.4%)	6 (66.7%)
Gender	23 (46.9%)	15 (65.2%)
Smoking friends	17 (34.7%)	13 (76.5%)
Dissatisfaction with family	11 (22.4%)	6 (54.5%)
Highly emotional environment	8 (16.3%)	3 (37.5%)
Divorce	5 (10.2%)	2 (40.0%)
Family disputes	12 (24.5%)	7 (58.3%)
History of school and family escape	3 (6.1%)	1 (33.3%)
Curiosity about smoking experience	9 (18.4%)	6 (66.7%)
Social problems	7 (14.3%)	2 (28.6%)
Low parental education level	10 (20.4%)	4 (40.0%)
Low family economic level	15 (30.6%)	2 (13.3%)

## Abbreviations

CI: Confidence interval
JBI: Joanna Briggs Institute
PRISMA: Preferred Reporting Items for Systematic Reviews and Meta-Analyses
SID: Scientific Information Database
WHO: World Health Organization
